# Global and Regional Prevalence and Outcomes of COVID-19 in People Living with HIV: A Systematic Review and Meta-Analysis

**DOI:** 10.3390/tropicalmed7020022

**Published:** 2022-02-03

**Authors:** Tope Oyelade, Jaber S. Alqahtani, Ahmed M. Hjazi, Amy Li, Ami Kamila, Reynie Purnama Raya

**Affiliations:** 1 Institute for Liver and Digestive Health, Division of Medicine, University College London, London NW3 2PF, UK; 2 Department of Respiratory Care, Prince Sultan Military College of Health Sciences, Dammam 34313, Saudi Arabia; jaber.alqahtani.18@alumni.ucl.ac.uk; 3 Centre for Haematology, Department of Inflammatory and Inflammation, College of Medicine, Imperial College London, London W12 0NN, UK; a.hjazi18@imperial.ac.uk; 4 Division of Surgery and Interventional Science, University College London, London NW3 2PS, UK; yutong.a.li@ucl.ac.uk; 5 Faculty of Science, Universitas ‘Aisyiyah Bandung, Bandung 40264, Indonesia; amikamila.unisabdg@gmail.com (A.K.); reynie.raya.18@ucl.ac.uk (R.P.R.); 6 Institute for Global Health, Faculty of Population Health Sciences, University College London, London NW3 2PF, UK

**Keywords:** COVID-19, HIV, public health, pandemic, infectious disease

## Abstract

Background: The relationship between HIV (human immunodeficiency virus) and COVID-19 clinical outcome is uncertain, with conflicting data and hypotheses. We aimed to assess the prevalence of people living with HIV (PLWH) among COVID-19 cases and whether HIV infection affects the risk of severe COVID-19 or related death at the global and continental level. Methods: Electronic databases were systematically searched in July 2021. In total, 966 studies were screened following the Preferred Reporting Items for Systematic Reviews and Meta-Analyses guidelines. Narratives were synthesised and data pooled for the global and continental prevalence of HIV–SARS-CoV-2 coinfection. The relative risks of severity and mortality in HIV-infected COVID-19 patients were computed using a random-effect model. Risk of bias was assessed using the Newcastle–Ottawa score and Egger’s test, and presented as funnel plots. Results: In total, 43 studies were included involving 692,032 COVID-19 cases, of whom 9097 (1.3%) were PLWH. The global prevalence of PLWH among COVID-19 cases was 2% (95% CI = 1.7–2.3%), with the highest prevalence observed in sub-Saharan Africa. The relative risk (RR) of severe COVID-19 in PLWH was significant only in Africa (RR = 1.14, 95% CI = 1.05–1.24), while the relative risk of mortality was 1.5 (95% CI = 1.45–2.03) globally. The calculated global risk showed that HIV infection may be linked with increased COVID-19 death. The between-study heterogeneity was significantly high, while the risk of publication bias was not significant. Conclusions: Although there is a low prevalence of PLWH among COVID-19 cases, HIV infection may increase the severity of COVID-19 in Africa and increase the risk of death globally.

## 1. Introduction

The 2019 coronavirus (COVID-19) pandemic caused by SARS-CoV-2 remains a global public health challenge that has affected over 186 million people and caused over 4 million deaths globally [1]. While most cases of COVID-19 are clinically mild or asymptomatic, older age and certain underlying illness, such as cardiovascular, respiratory, and digestive diseases, have been reported to increase the risk of severe COVID-19 cases or death [2,3,4]. Such comorbidities are associated with an increased fatality rate and present a challenge for intensive care management of COVID-19 patients [5,6].

Human immunodeficiency virus (HIV) belongs to a genus of zoonotic lentiviruses that causes acute immune deficiency syndrome (AIDS) [7]. Data from the Joint United Nations Programme on HIV/AIDS (UNAIDS) puts the number of people living with HIV (PLWH) at 38 million globally, with 1.5 million new infections in 2020 and about 6 million people unaware of their HIV infection status [8]. Accordingly, the number of PLWH is projected to increase due to treatment availability and the associated reduction in AIDS-related deaths [9].

HIV is associated with dysregulation of the immune system, which predisposes patients to opportunistic infectious diseases [10]. Indeed, most HIV-related deaths have been linked to secondary infections and abnormal inflammatory response resulting from AIDS [11]. This is especially so in patients with uncontrolled HIV replication, a high viral load and a low CD4/CD8 count. Giving the immune-compromised state of most PLWH and the increased possibility of secondary dysfunctions, an increased risk of infection, severity and death due to COVID-19 may be expected. However, an attenuated immune response may also protect against the cytokine release storm and the corresponding acute respiratory distress syndrome (ARDS) linked with severe SARS-CoV-2 infection or the associated mortality [12]. Indeed, various ARTs (antiretroviral therapies) used for HIV treatment were also proposed as candidates for the treatment of SARS-CoV-2 infection in the early stage of the COVID-19 pandemic, and there have been initial hypotheses that HIV patients undergoing ART or pre-exposure prophylaxis (PrEP) may have collateral immunity to COVID-19. However, most findings showed no significant positive effect of ART on COVID-19 infection or outcomes compared with standard care [13,14,15]. Further, a study by Ayerdi et al. assessing whether ART or PrEP usage had a preventative effect on the seroprevalence and clinical course of COVID-19 among men who have sex with men and transgender women found no significant positive effect [16].

To understand the relationship between COVID-19 and HIV infection, previous systematic reviews and meta-analysis have been published, including the studies by Mellor et al. and Hariyanto et al., which both found increased risks of severe COVID-19 and mortality in PLWH compared with HIV-negative COVID-19 cases [17,18]. Moreover, a systematic review by Ssentongo et al., involving 22 reports from Africa, Asia, Europe and North America, showed an increased risk of mortality from COVID-19 in PLWH [19]. On the contrary, the study by Gao et al., reported no significant increase in the risk of severe COVID-19 or related death due to HIV infection [20]. This was corroborated by the study by Lee et al. involving 643,018 PLWH, which reported no significant increase in the risk of adverse outcomes of COVID-19 in PLWH [21]. Hence, the association between HIV infection and COVID-19 outcomes remain unclear, with sparse and conflicting reports.

Aside from the heterogeneity from the established difference in the epidemiology of HIV between countries and continents, variability also exists in the treatment and management of HIV infection, as well as the behaviour of PLWH in various regions of the world. These, amongst other factors, determine the rate of spread, as well as the availability and uptake of preventative and treatment measures for HIV [22]. The disruption to clinical care of various chronic diseases due to the diversion of medical resources to manage the increasing COVID-19 cases around the world at the peak of the COVID-19 pandemic further contributes to the increased global variability in the clinical course of COVID-19 in PLWH [23]. This review aimed to provide an updated insight into the global and continental prevalence of PLWH among COVID-19 cases and the potential risk of severe COVID-19 and death associated with HIV infection by conducting a meta-analysis of HIV-positive and HIV-negative COVID-19 patients grouped by continents.

## 2. Methods

The protocol of this systematic review was registered prospectively to PROSPERO (CRD42021264151). Following the Preferred Reporting in Systematic Reviews and Meta-Analyses (PRISMA) guidelines [24], the Medline and Embase databases were searched on 2 July 2021 using keywords and MeSH terms (Figure S1). Further, a search of preprint databases (www.medrxiv.org and www.preprint.org, accessed on 2 July 2021) was also performed on the 2nd of July 2021 because of the rapidly developing nature of the topic. The search of preprint databases did not follow a systemic search strategy because concatenation was not feasible. However, the MeSH terms for HIV and COVID-19 as described in the supplemental figure (Figure S1) were combined consecutively and the resulting studies’ titles were screened. Studies retrieved from the databases were imported into EndNote software, and duplicate records were removed. The resulting duplicate-free studies were then uploaded to Rayyan software, and title, abstract and whole-text screening was carried out (RPR, AK). Reference screening of the included studies and relevant peer-reviewed previously published reports was also performed to retrieve studies that were not covered by the search strategy. The reference search did not include any search strategy and involved identifying references that were cited within published studies that that were published on the topic. 

### 2.1. Inclusion and Exclusion Criteria 

The inclusion/exclusion of studies followed the PECO (Population, Exposure, Comparison and Outcome) model [25]. We included only studies that presented the clinical characteristics and/or composite endpoints of COVID-19 patients and reported the proportion of these patients with a pre-existing HIV infection. Studies that included both hospitalized and community-based COVID-19 patients were also included to understand the overall prevalence of HIV infection as a comorbidity in COVID-19 cases, irrespective of the hospitalization status. However, only studies with clinically confirmed outcomes of COVID-19 cases were included in the meta-analysis for the risk of severity and mortality associated with HIV comorbidity. Studies that combined HIV and other immunosuppressive diseases and conditions (cancer, congenital, or medically induced), non-English language publications, reviews, case reports, qualitative studies, editorials, and studies including only patients that died from COVID-19 were excluded. Studies that also focused on only HIV patients coinfected with SARS-CoV-2 were included in the systematic review but not in the meta-analysis. This is because most of these studies focused only on HIV patient recruitment and did not provide a comparative analysis of risk in patients without HIV. Moreover, studies focused on HIV patients alone may introduce bias in recruitment, which may target PLWH more. For studies in which suspected and confirmed COVID-19 cases were reported [26], we only synthesised the number of confirmed cases. In addition, studies including the same (duplicate) population of patients were identified and included in the systematic review [27,28]. However, only the latest study was included in the meta-analysis [27]. 

### 2.2. Data Collection

Two authors (RPR, AK) independently screened the titles and abstracts of potentially eligible studies, and conflicts were resolved through mediation by a third reviewer (TO). The full text of potential studies that were included from the abstract screening stage were fully read and assessed against the inclusion/exclusion criteria. 

### 2.3. Data Extraction and Analysis 

The authors and year of publication, the study design and period, the country of study, the sample size of COVID-19 cases and the proportion that had HIV as a comorbidity, as well as the clinical outcomes of both groups (PLWH and non-HIV COVID-19 patients) were extracted into a table. Clinical outcomes identified were the severity of the COVID-19 and death linked to infection with the SARS-CoV-2 virus. Severe COVID-19 was defined as a prolonged hospital stay, ICU admission and/or need for mechanical ventilation (MV) as a result of reduced oxygen saturation (<90% of room air), and a respiratory rate of >30 breaths/minute and signs of severe respiratory distress according to the WHO recommendations [29]. Analysis was performed using Stata/MP 17; prevalence was calculated by the “metaprop” procedure using the random effect model. Forest plots were used to present the pooled prevalence of PLWH in COVID-19 cases grouped by the continent of study. Continent-grouped effect sizes (95% confidence intervals, CIs) and the test results of between-study heterogeneity (I^2^ statistic, *p*-value) were also computed using the random effect model. The “metan” procedure was used to assess the risk of severity and mortality in PLWH-COVID-19 patients compared with the general population in the included studies, and the risk ratios were grouped by continents to further assess the intercontinental variation in these risks. All meta-analysis was performed using the random effect model, which is more robust to the between-study heterogeneity expected in the pooled studies, which were performed in different regions of the world with different health, socio-economic and research standards.

### 2.4. Quality Assessment

A modified version of the Newcastle–Ottawa Score (NOS) was used to assess the risk of bias in the included studies [21]. This includes 3 domains and 9 questions scored accordingly with a star. The “selection” domain assessed the randomness and multicentre involvement in the selection of the study population, as well as the sample size. The multicentre recruitment of patients was scored because this design provides better quality data and more generalizable results because more centres better represent the study population than a single centre [30]. A sample size of ≥100 was decided on the basis of previous studies’ estimates of ~1% prevalence of HIV infection in COVID-19 cases [21,31]. The standard ascertainment of COVID-19 and HIV were also assessed against the WHO guidelines [32,33]. Finally, the follow-up time (≥2 weeks), mode of outcome confirmation and whether all patients were accounted for were also assessed (Table S1). Studies with ≥5 stars (>50%) were considered unbiased. To further assess publication bias in the studies pooled for prevalence and the risk of severity and mortality in COVID-19-infected PLWH, funnel plots and the Egger test were computed using the “metafunnel” and “metabias” procedures respectively in STATA. Statistical significance was set at 95% (*p* < 0.05).

We also performed a “leave-one-out” sensitivity analysis using the “meta forestplot, leaveoneout” procedure in STATA to assess whether any of the studies included in the computation of the prevalence and risk ratios were producing misleading and exaggerated effect sizes. The procedure usually performs multiple computations by consecutive removal of one study at each analysis and presenting the effect sizes generated in a forest plot.

## 3. Results

**Figure 1:**
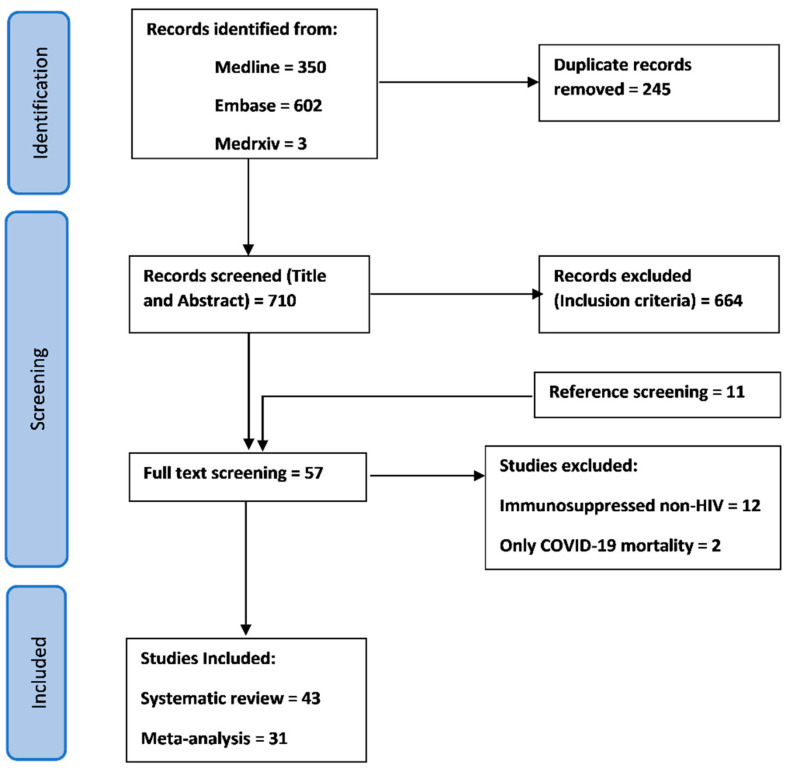
Global and regional prevalence and outcomes of COVID-19 in people living with HIV: A systematic review and meta-analysis according to the Preferred Reporting for Systematic Reviews and Meta-analyses diagram.

The systematic search of databases including preprints and the reference search generated an initial total of 955 studies, including 245 duplicates, to give a total of 710 studies. Initial title and abstract screening led to the exclusion of 664 studies, followed by full-text review of the 46 potentially eligible studies. Full-text screening resulted in further exclusion of 14 studies, while screening of the references of relevant studies resulted in 11 eligible studies to give a total of 43 studies which satisfied the inclusion/exclusion criteria (Figure 1).

### 3.1. General Description of the Studies Included

The 43 studies in the systematic review included 692,032 COVID-19 cases, of which 9097 (1.3%) were PLWH. The sample sizes of the included studies ranged from 20 to 378,248, with data from 15 countries across five continents. Overall, 27 of the studies were retrospectively performed, with 11 prospective studies, 2 descriptive studies and 3 case series (Table 1). Of the included studies, 10 assessed only PLWH coinfected with SARS-CoV-2 and were excluded from further analysis. Another study was excluded because it involved selective matching of PLWH and non-HIV COVID-19 cases [34], and one study [28] that was conducted on the same cohort of patients was excluded in favour of the more recently published one [27]. The risk of bias assessment showed low bias in the included studies, with 86% (37/43) of the studies below the bias threshold (Table S1). 

### 3.2. Prevalence of PLWH among COVID-19 Cases

**Figure 2:**
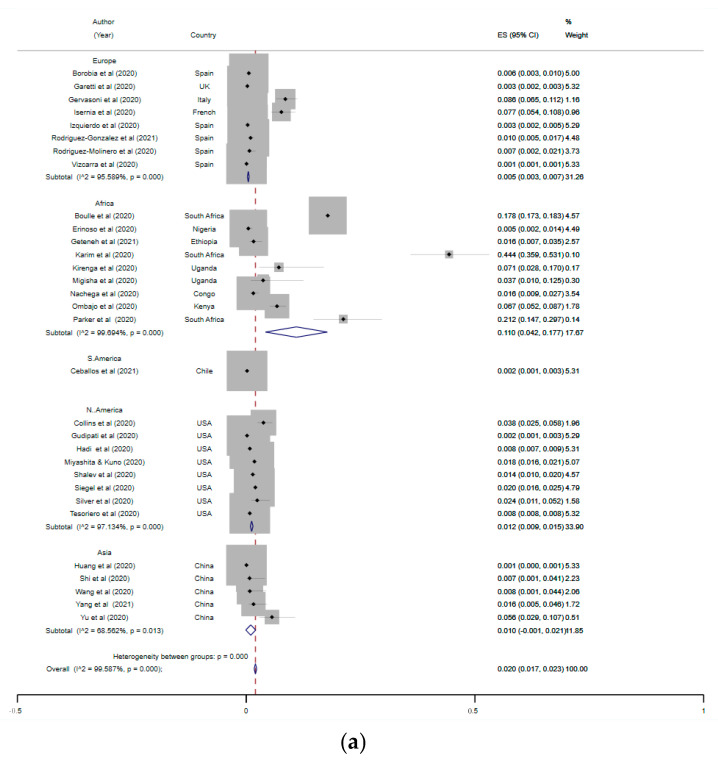

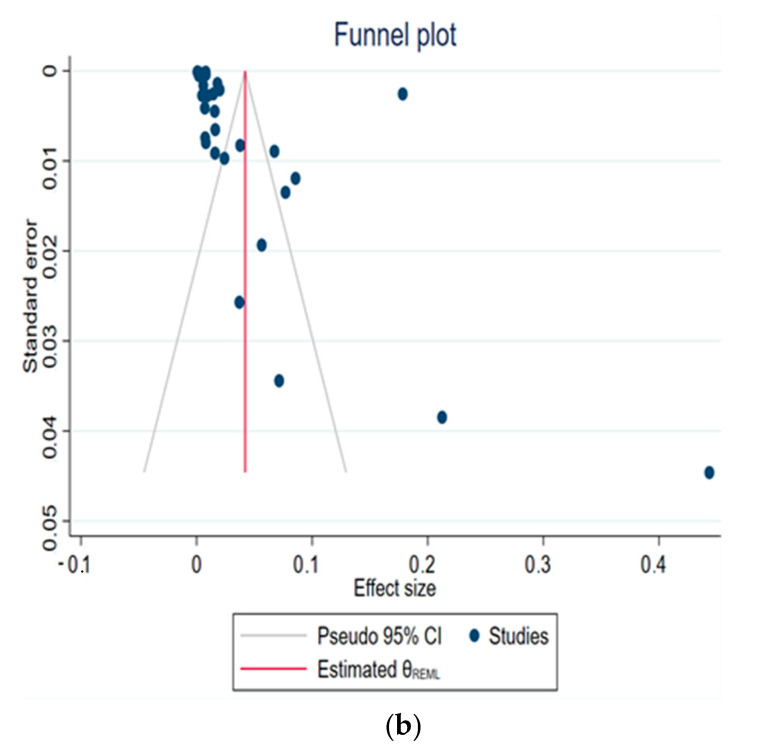
(**a**) Pooled prevalence of PLWH among COVID-19 cases. The red dotted line represents the overall effect size. The lateral edges of the blue diamonds represent the limits of the 95% confidence intervals (ES: effect size; CI: confidence interval). (**b**) Funnel plot of studies pooled for the prevalence of PLWH among COVID-19 cases (ES: effect size; se: standard error).

Of the 43 studies included, 10 studied COVID-19 infections in only PLWH, while one study (68) was designed as a case–control study and was excluded from the meta-analysis [39,40,42,47,48,50,55,56,62,69]. Two studies were identified as duplicate data [27,28] and only the most recent version [27] was included. Nine of the studies analysed for prevalence were conducted in Africa, with eight each conducted in Europe and North America. The global pooled prevalence of PLWH among COVID-19 cases was 2% (95% CI = 1.7–2.3%, *p* < 0.001) while at the continental level, the pooled prevalence for Europe and North America was 0.5% and 1.2%, respectively. Moreover, 75% (6/8) of the studies from the USA included in the meta-analysis were conducted in the states (New York and Georgia) with the highest HIV infection rates, according to recent data [73], which may explain the higher prevalence in North America compared with Europe. The pooled prevalence of studies from Africa was expectedly the highest at 11% (95% CI, 4–18%), while that of continental Asia was 1% (95% CI, −0.1–2%). The negative 95% CI in the pooled prevalence of PLWH in COVID-19 shown by studies from Asia may be associated with the random effect model used for intercontinental pooling of studies. However, the prevalence remained the same (1%) and there was no significant between-study heterogeneity when the analysis was performed for studies from Asia separately (Figure S5). Further, 67% (6/9) of the studies from Africa were from East and Southern Africa, the region with over half (55%) of the total global HIV infections according to the 2021 estimate [74]. The variation in the prevalence of HIV infection in this study is illustrative of the current global epidemiology of HIV, whereby more than two-thirds of PLWH are currently in Sub-Saharan Africa [75]. Moreover, the overall between-study heterogeneity was significantly high (I^2^ = 99.7%, *p* < 0.001; Figure 2a) and this was expected, due to the variation in global distribution of PLWH. Publication bias in the pooled studies was further assessed by computing a funnel plot and Egger’s test, which was significant (T (95% CI) = 2.17 (0.39–12.18), *p* = 0.04; Figure 2b). The sensitivity test showed that there was no significant reduction in heterogeneity following successive omission of studies, as the global pooled prevalence still ranged between 3% and 4% (Figure S2).

### 3.3. Severity of COVID-19 in PLWH

**Figure 3:**
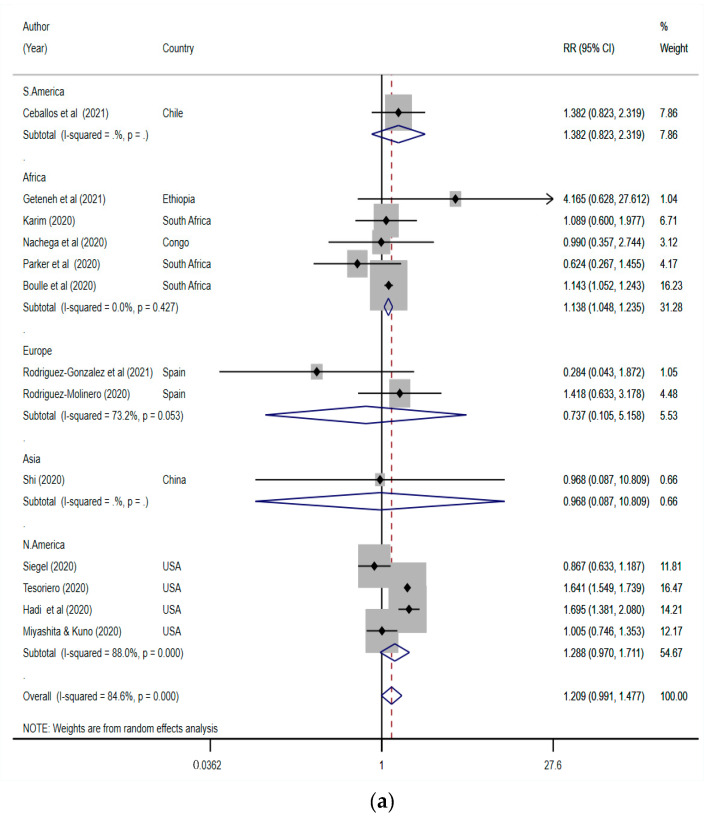

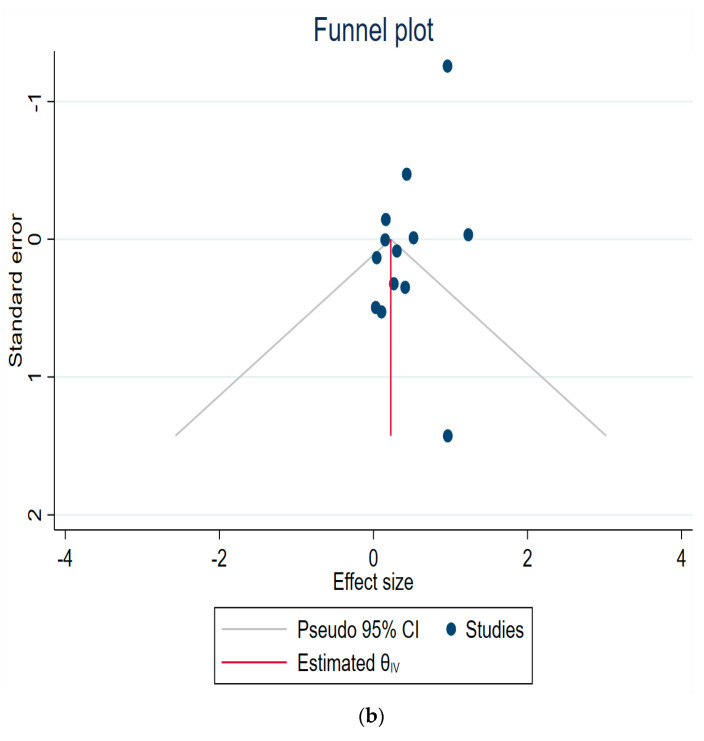
(**a**) Forest plot of studies pooled for the risk of severe COVID-19 in PLWH. The red dotted line represents the overall effect size/risk ratio. The lateral edges of the blue diamonds represent the limits of the 95% confidence intervals (RR: risk ratio; CI: confidence interval). (**b**) Funnel plot of studies pooled for the risk of severe COVID-19 in PLWH (CI: confidence interval).

Thirteen studies presented data on the severity of COVID-19 in PLWH and non-HIV patients, and were analysed to determine the risk of severity in PLWH compared with non-HIV COVID-19 patients [36,37,44,46,53,58,59,61,63,64,66,67,68]. These studies included a total of 485,540 COVID-19 cases, of whom 7768 (1.6%) were PLWH. Overall, five, four and two of the pooled studies were conducted in Africa, the USA and Europe, respectively. The pooled global risk ratio was not significant and showed that PLWH may not be at risk of developing severe COVID-19 (RR (95% CI) = 1.21 (0.99–1.48); *p* = 0.477; Figure 3a). However, this result was very close to significance, and including more data in the future may provide further insight into the relationship between HIV infection and the severity of COVID-19. Indeed, this lack of significance was true for both Europe and USA, regions associated with better prevention and management of HIV infections. However, the risk for severe COVID-19 among PLWH from Africa was found to increase by 14% (RR (95% CI) = 1.14 (1.05–1.24) compared with non-HIV COVID-19 patients. Moreover, while the overall heterogeneity was significantly high (85%, *p* < 0.001), there was no between-study variation in the studies from Africa (I^2^ = 0%, *p* = 0.43). Indeed, the funnel plot showed no publication bias and the Egger’s test showed no small study effect (T (95% CI) = −1.32 (−3.02 to 0.75), *p* = 0.21; Figure 3b). The sensitivity test showed that leaving out some studies produced a significant result (Figure S3). However, doing so did not significantly improve the between-study heterogeneity of the results.

### 3.4. Mortality of PLWH Coinfected with SARS-CoV-2

**Figure 4:**
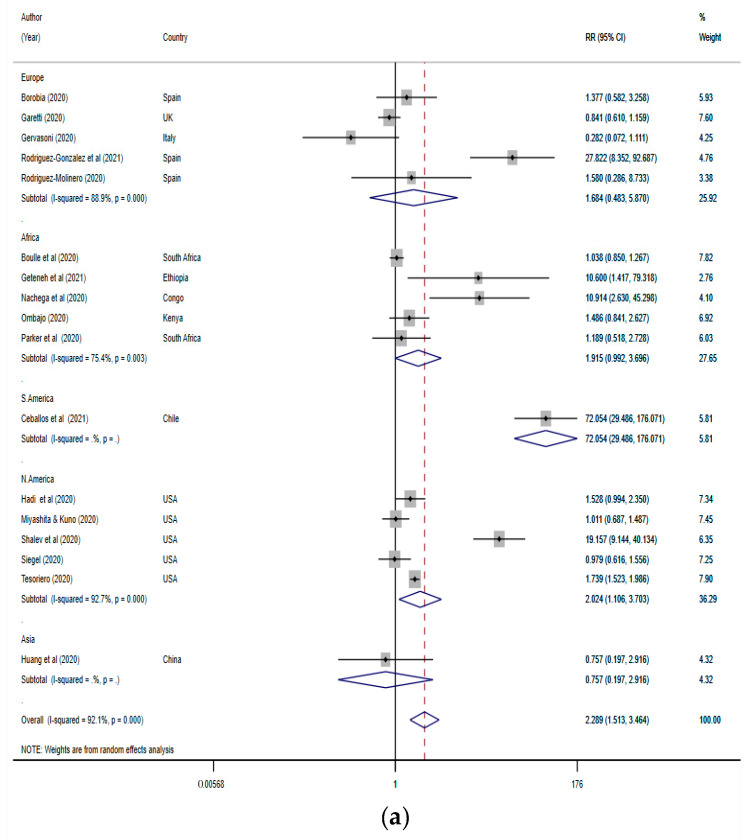

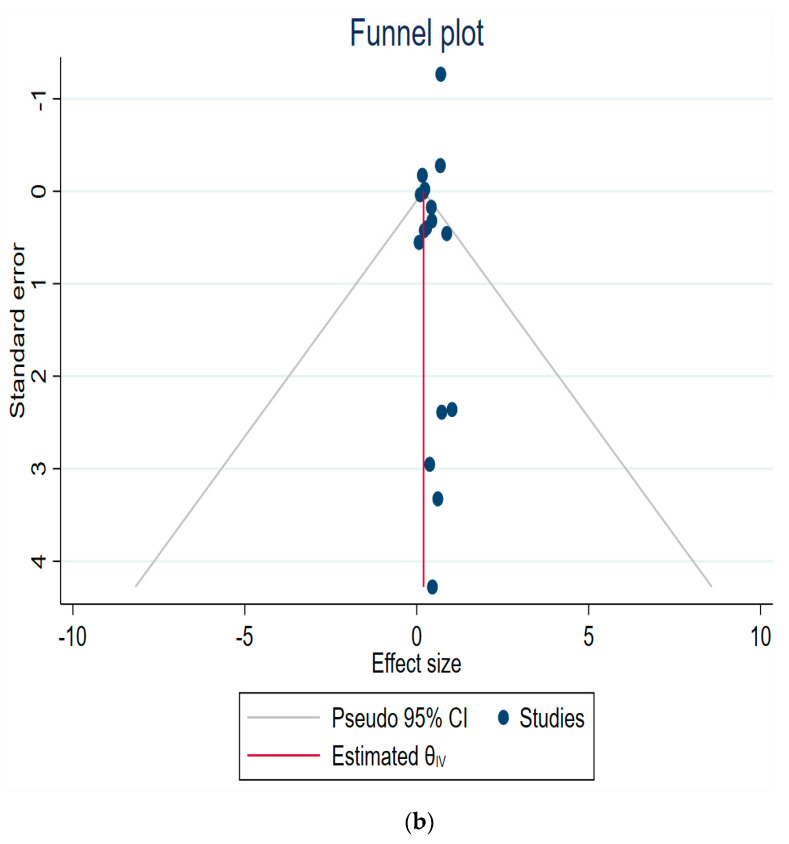
(**a**) Forest plot for COVID-19 mortality in PLWH. The red dotted line represents the overall effect size/risk ratio. The lateral edges of the blue diamonds represent the limits of the 95% confidence intervals (RR: risk ratio; CI: confidence interval). (**b**) Funnel plot of studies pooled for COVID-19 mortality in PLWH (CI: confidence interval).

In total, 17 studies were included in the assessment of the risk of mortality from COVID-19 in PLWH compared with non-HIV COVID-19 patients [27,35,36,37,43,44,46,49,58,59,60,61,63,64,65,67,68]. The 17 studies had 588,960 COVID-19 cases, including 8013 (1.4%) PLWH. Five each of the analysed studies were conducted in Africa, Europe, and North America. The meta-analysis results showed that HIV infection increased the risk of death from COVID-19 by 2.3-fold globally (RR (95% CI): 2.29 (1.51–3.46); Figure 4a) compared with COVID-19 patients without HIV. On the regional level, there was no significantly increased risk of COVID-19 mortality in PLWH in Africa or Europe. However, a twofold increase in risk of mortality was observed in the USA according to the studies included. Despite this difference in regional risk ratios, the computed funnel plot showed no publication bias (Figure 4b), and Egger’s test showed no small study effect in the included studies (T (95% CI) = 1.22 (−1.13 to 4.17)). The sensitivity test showed that the significance was not influenced by the removal of any of the included studies (Figure S4). 

## 4. Discussion

This study provides an updated systematic assessment of the prevalence and clinical outcomes of COVID-19 in PLWH compared with the general population. The results were stratified to present the prevalence of PLWH among COVID-19 cases as well as the clinical outcomes at both the global and regional level. To the best of our knowledge, this is the first systematic review and meta-analysis dedicated solely to understanding the clinical outcome of SARS-CoV-2-infected patients who had HIV infection on both the global and continental levels. Our method of analysis considered the regional variation in the prevalence, prevention and management of HIV infection in the included continents. We found a significantly lower global prevalence of PLWH in COVID-19 cases (2%) compared with other comorbidities such as cardiovascular disease and obesity. This is consistent with previous reports which estimated the prevalence of HIV coinfection at 1–2% of COVID-19 patients either admitted to the hospital or in the general population [19,76]. Furthermore, while the proportion was below 2% in Europe, North America and Asia, the prevalence of PLWH in COVID-19 cases was found to be relatively high in Africa (11%). This is reflective of the global epidemiology of HIV, whereby more than half of the global cases are located within continental Africa. Interestingly, 75% of the studies from Africa were performed in the East and Southern Africa region, which accounts for over 54% of the global HIV cases [74]. Our finding is consistent with earlier systematic reviews, which showed a similar prevalence of HIV–SARS-CoV-2 coinfection cases [19,21].

Our result also showed that PLWH may not be at a relatively higher risk of severe COVID-19, defined by admission to intensive care units or the need for mechanical ventilation, at the global level. Interestingly, this lack of an association between HIV infection and COVID-19 severity held true in Europe and the United States, but not in Africa. We found a 15% increase in the risk of severe COVID-19 for PLWH in Africa. Moreover, 60% (3/5) of the studies analysed for the risk of severe COVID-19 in Africa were conducted in South Africa, and all studies originated from sub-Saharan Africa, a region associated with a high HIV infection rate and poorer antiretroviral treatment (ART) availability [74]. Furthermore, we found a twofold increase in the relative risk of death from COVID-19 in PLWH at the global level. However, only the North American (United States) continent showed a significant risk of mortality (twofold) among the regions computed. Moreover, most studies within the USA were conducted in Georgia and New York, both of which are among the top 10 states with the highest HIV infections and that were hardest hit by the COVID-19 pandemic [1,77].

Importantly, our findings corroborate some previous reports on the potential risk of a severe clinical course of COVID-19 in PLWH. Specifically, various meta-analyses were conducted on the difference in risk of severe COVID-19 between HIV-positive and HIV-negative patients with SARS-CoV-2 infection, whereby the risk of severe COVID-19 and mortality were found to be associated with HIV status [17,19]. However, other reports have been conflicting, with no difference in the risk of severe COVID-19 or death between HIV-positive and HIV-negative patients [19,21], with one report proposing a protective effect of HIV infection against COVID-19 [78]. Further, Liang et al. reported that HIV infection was not related to poorer COVID-19 outcomes, and concluded that any risk observed in HIV–SARS-CoV-2 coinfection may be related to the presence of concomitant comorbidities, which may be common in patients with undiagnosed or untreated HIV infection [31]. Lee et al. also reported no relationship between HIV infection and the clinical outcome of COVID-19 following a systematic review of 643,018 PLWH [21]. However, a systematic review by Mellor et al. involving a meta-analysis of five studies showed that PLWH had an increased risk of mortality due to COVID-19 compared with HIV-negative patients [18]. Further, a meta-analysis and meta-regression of PLWH in COVID-19 cases performed by Hariyanto et al. found that an increased risk of mortality was only significant in the studies from Africa and the United States after controlling for age, CD4 cell count or anti-retroviral treatment [16]. The results of this study support our findings regarding the significant increase in the risk of death due to COVID-19 in PLWH from the United States. However, while the risk of mortality was not significant in Africa, our results were close to statistical significance (0.992–3.696; Figure 4a); more studies may improve this result in future analysis. Notably, most of the previous systematic reviews with or without a meta-analysis were carried out earlier in the COVID-19 pandemic period and included case reports with fewer studies included in the meta-analyses.

The observed increased risk of severe illness (Africa) and death (globally) from COVID-19 in these studies may be attributed to the interplay between several factors. Firstly, the availability of effective HIV management tools in developed countries means that PLWH now live longer in these regions [79]. Increased age is associated with senescence of the natural immune system, which may combine with other immune-dampening features of chronic, untreated HIV infection to increase the risk of severity and death from COVID-19. Moreover, PLWH, especially those with undiagnosed or uncontrolled infections, low CD4 count, opportunistic infections and a high viral load, may present with severe COVID-19 and are at higher risk of death [80]. Aside from CD4 and CD8 T-cell activation, effective and early immunoglobin G (IgG) generation results in effective SARS-CoV-2 clearance and improves clinical outcomes [81]. However, uncontrolled HIV replication may trigger increased CD8 T-cell activation, inflammation, T-cell exhaustion and dysfunction in B-cells’ activities [82,83]. The combined breakdown of B- and T-cell functions resulting from natural immune system exhaustion may not only result in poorer COVID-19 outcomes but also compromise the efficacy of vaccines in PLWH. Indeed, the response to and efficacies of various vaccines, including hepatitis B, pneumococcal, influenza vaccines, have been shown to be diminished in PLWH, and repeated or modified vaccine administration has been recommended [84,85,86]. However, evidence on COVID-19 vaccine efficacy in PLWH is scarce, and vaccination of HIV-positive and -negative people remains similar. Effective ART can attenuate most of the immune dysregulation resulting from uncontrolled HIV infection and replication, and is highly recommended. However, undiagnosed HIV infection and low uptake of ART, both of which are prevalent in Africa, may predispose patients to poorer COVID-19 clinical outcomes [74].

Moreover, the prevention (sensitisation and pre-exposure prophylaxis), diagnosis and management (ART) of HIV and other chronic diseases have been affected by the global shift in medical resources to contain the COVID-19 pandemic [23]. This shift has been suggested to be a contributory factor to the susceptibility of affected groups to severe COVID-19 and death [87,88,89]. Expectedly, the disruption to healthcare systems, especially HIV clinics, and the downstream effect have been relatively worse in developing countries, possibly resulting in worse outcomes for PLWH coinfected with COVID-19 [90]. However, more data will be needed to establish the extent of these disruptions in regions already behind in the fight against HIV, and the contributory effects of other established confounders that may drive the clinical outcome of COVID-19 in patients with pre-existing HIV infection.

Put together, our result show that while the risk of severity illness and death due to COVID-19 increased respectively in Africa and globally, the mechanistic link between HIV infection and the clinical course of COVID-19 may be more complex than previously thought. Firstly, the regional aggregation performed in this study showed that the prevalence of PLWH in COVID-19 cases is best translated in the context of the current global epidemiology of HIV infections. Indeed, the variability introduced by the differences in regional HIV infection rates made estimation of the global prevalence of HIV–SARS-CoV-2 coinfection less reliable if not controlled for the regional prevalence of HIV. Secondly, there are complex, hardly resolvable confounders when assessing the relationship between HIV infection and COVID-19 outcomes, including age, sex, treatment with ART, race, region, immune state of the patient, number and types of comorbidities and the duration of comorbidities, among other factors, and we recommend further research to clarify this topic in the context of these and other confounders. Indeed, Bhaskaran et al. [91] controlled for age, sex, ethnicity, comorbidities, and time in a population of COVID-19 patients within the United Kingdom. However, the regional differences in prevalence, prevention techniques and clinical management of both HIV and COVID-19, as well as various social-economic factors, mean that their findings may not reflect the situation outside the United Kingdom.

This study has several limitations. Firstly, some of the included studies were case series reporting only PLWH coinfected with SARS-CoV-2. However, such studies were excluded from the prevalence analysis. Moreover, the random effect model was used to reduce the effect of variations in the experimental design on the computed results. Secondly, most studies did not report the distributions of comorbidities, race, age, CD4 and CD8 counts, duration of HIV infection or ART use, among other confounders, in the studied groups. Thus, we could not adjust for these parameters in this study. Moreover, some studies did not report the clinical outcomes (death and severity) of COVID-19 in both PLWH and patients who were HIV-negative, and these could not be included in the relative risk computation. However, Egger’s test and the funnel plots showed that there was no publication bias in the analysed records, while the sensitivity analysis also showed no exaggeration of the result due to individual studies. In addition, overlapping data are generally a major limitation in systematic reviews and meta-analyses, which may also limit the interpretation of this study’s results [92]. However, records found to be overlapping were excluded in favour of the most recent report. Finally, our database search was restricted to full-text studies alone. Thus, more relevant studies may be available and should be considered for future analyses of this topic.

Our findings have several clinical and research implications. First, it further widens the body of evidence by including more recent and high-quality studies to report that HIV infection may increase the risk of severe COVID-19 and death, and which regions of the world present with more risk to PLWH. Secondly, we show that the risks of severe COVID-19 and death in PLWH varied between continents and may reflect a complex interplay of concomitant contributory factors, which may need to be controlled for to better understand the direct or indirect effects of HIV infection on COVID-19 outcomes. Moreover, the prevalence of HIV–SARS-CoV-2 coinfection is best interpreted in the context of the varied global epidemiology of HIV infection in various regions of the world. Considering the complex effect of HIV infection on the host immune system as well as the dependence of vaccine efficacy on the immune response, future studies should assess the COVID-19 vaccine’s pharmacokinetics in HIV-positive patients to decide whether PLWH coinfected with SARS-CoV-2 may benefit from certain types of vaccines, prioritisation, or repeated inoculations.

## Figures and Tables

**Table 1:** General characteristics of included studies.

Study Name (Year)	Country	Type of Study	Study Participants	Sample Size (M; F; T)	Age (Mean ± SD or Median, Range)	PLWH	PLWH Surviving	PLWH Non-Surviving	PLWH Severe	PLWH Non-Severe
Borobia et al. (2020) [35]	Spain	Retrospective	COVID-19 cases	2226 (M = 1074; F = 1152)	61 (IQR 46–78)	13	9	4	NR	NR
Boulle et al. (2020) [36]	South Africa	Retrospective	COVID-19 cases	22,308 (NR)	(NR)	3978	3863	115	601	3262
Ceballos et al. (2021) [37]	Chile	Prospective	COVID-19 cases	18,321 (M = 10300; F = 8021)	NR	36	31	5	11	25
Collins et al. (2020) [38]	USA	Case series	COVID-19 cases	530 (NR)	NR	20	17	3	3	17
Del Amo et al. (2020) [39]	Spain	Prospective	HIV–SARS-CoV-2 coinfected cases	236 (M = 204; F = 32; all PLWH)	NR	236	216	20	15	221
Di Biagio et al. (2020) [40]	Italy	Prospective	HIV–SARS-CoV-2 coinfected cases	69 (NR; all PLWH)	NR	69	62	7	4	58
Docherty et al. (2020) [28]	UK	Prospective	COVID-19 cases	20,133 (M = 12,068; F = 8065)	73 (IQR 58–62)	83	37	23	NR	NR
Erinoso et al. (2020) [41]	Nigeria	Retrospective	COVID-19 cases	632 (M = 385, F = 247)	40.1 (SD ± 13.9)	3	NR	NR	NR	NR
Etienne et al. (2020) [42]	France	Prospective	HIV–SARS-CoV-2 coinfected cases	54 (M = 33; F = 21; all PLWH)	54 (range 47–60)	54	53	1	19	35
Geretti et al. (2020) [27]	UK	Prospective	COVID-19 cases	47,592 (NR)	NR	122	75	30	NR	NR
Gervasoni et al. (2020) [43]	Italy	Retrospective	COVID-19 cases	549 (NR)	51 ± 11	47	45	2	2	34
Geteneh et al. (2021) [44]	Ethiopia	Retrospective	COVID-19 cases	372 (M = 279; F = 93)	30 (5–85)	6	5	1	1	5
Gudipati et al. (2020) [45]	USA	Case series	COVID-19 cases	7372 (NR)	NR	14	11	3	2	12
Hadi et al. (2020) [46]	USA	Retrospective	COVID-19 cases	50,167 (NR)	NR	404	384	20	78	326
Harter et al. (2020) [47]	Germany	Retrospective	HIV–SARS-CoV-2 coinfected cases	33 (M = 30; F = 3)	48 (range 26–82)	33	29	3	8	25
Ho et al. (2021) [48]	USA	Retrospective	HIV–SARS-CoV-2 coinfected cases	93 (M = 67; F = 23, T = 3; all PLWH)	58 (range 52–65)	93	74	19	19	74
Huang et al. (2020) [49]	China	Retrospective	COVID-19 cases	50368 (NR)	NR	35	33	2	15	20
Inciarte et al. (2020) [50]	Spain	Prospective	HIV–SARS-CoV-2 coinfected cases	53 (NR)	NR	53	51	2	10	43
Isernia et al. (2020) [51]	France	Case series	COVID-19 cases	390 (NR)	NR	30	24	2	4	24
Izquierdo et al. (2020) [52]	Spain	Retrospective	COVID-19 cases	10504 (M = 5519; F = 4984)	58.2 ± 19.7	34	NR	NR	1	33
Karim et al. (2020) [53]	South Africa	Retrospective	COVID-19 cases	124 (M = 30; F = 94)	45 (IQR, 35.0–57.4)	55	NR	NR	16	39
Kirenga et al. (2020) [54]	Uganda	Prospective	COVID-19 cases	56 (M = 38; F = 18)	34.2 ± 15.5	4	4	0	NR	NR
Liu et al. (2020) [55]	China	Retrospective	HIV–SARS-CoV-2 coinfected cases	20 (M = 5; F = 15)	46.5 (IQR, 39.3–50.5)	20	19	1	3	17
Maggiolo et al. (2021) [56]	Italy	Prospective	HIV–SARS-CoV-2 coinfected cases	55 (M = 44; F = 11)	54 (49–58)	55	51	4	11	44
Migisha et al. (2020) [57]	Uganda	Retrospective	COVID-19 cases	54 (M = 34; F = 20)	NR	2	2	0	0	2
Miyashita and Kuno (2021) [58]	USA	Retrospective	COVID-19 cases	8912 (NR)	NR	161	138	23	36	125
Nachega et al. (2020) [59]	Congo	Retrospective	COVID-19 cases	766 (M = 500; F = 262; unknown = 4)	34 ± 4.5	12	10	2	3	9
Ombajo et al. (2020) [60]	Kenya	Retrospective	COVID-19 cases	787 (M = 505; F = 282)	43 (range 0–109)	53	42	11	NR	NR
Parker et al. (2020) [61]	South Africa	Retrospective	COVID-19 cases	113 (M = 44; F = 69)	NR	24	18	6	5	19
Pujari et al. (2021) [62]	India	Retrospective	HIV–SARS-CoV-2 coinfected cases	86 (M = 66; F = 20)	45 ± 52.3	86	80	6	17	69
Rodriguez-Gonzalez et al. (2021) [63]	Spain	Retrospective	COVID-19 cases	1255 (M = 725; F = 530)	65 (range 51–77)	12	9	3	1	11
Rodriguez-Molinero et al. (2020) [64]	Spain	Prospective	COVID-19 cases	418 (M = 238; F = 180)	65.4 ± 16.6	3	2	1	3	0
Shalev et al. (2020) [65]	USA	Retrospective	COVID-19 cases	2159 (NR)	NR	31	23	8	2	29
Shi et al. (2020) [66]	China	Retrospective	COVID-19 cases	134 (M = 65; F = 69)	46 (IQR: 34–58)	1	1	0	0	1
Sigel et al. (2020) [67]	USA	Retrospective	COVID-19 cases	4402 (NR)	NR	88	70	18	18	70
Silver et al. (2020) (60)	USA	Retrospective	COVID-19 cases	249 (M = 110; F = 139)	59.6	6	NR	NR	NR	NR
Stoeckle et al. (2020) [34]	USA	Retrospective (case-control)	COVID-19 cases	120 (M = 96; F = 24)	60.5 (range 56.6–70.0)	30	24	2	4	NR
Tesoriero et al. (2021) [68]	USA	Descriptive	COVID-19 cases	378248 (M = 192,646; F = 183,319)	NR	2988	689	207	896	2092
Virata et al. (2020) [69]	USA	Retrospective	HIV–SARS-CoV-2 coinfected cases	40 (M = 20; F = 20)	NR	40	40	0	4	36
Vizcarra et al. (2020) [26]	Spain	Prospective	COVID-19 cases	61,577 (NR)	NR	51	44	2	6	45
Wang et al. (2020) [70]	China	Descriptive	COVID-19 cases	125 (M = 71; F = 54)	38.76 ± 13.799	1	1	0	NR	NR
Yang et al. (2021) [71]	China	Retrospective	COVID-19 cases	188	NR	3	NR	NR	NR	NR
Yu et al. (2020) [72]	China	Retrospective	COVID-19 cases	142 (M = 81; F = 61)	61.9 ± 12.4	8	NR	NR	NR	NR

M, male; F, female; T, transgender man/woman; SD, standard deviation; IQR, interquartile range; PLWH, people living with HIV; NR, not reported.

## Supplementary Materials

The following are available online at https://www.mdpi.com/article/10.3390/tropicalmed7020022/s1, Table S1: Quality Assessment; Figure S1: Search Strategy; Figure S2: An influence plot from a “leave-one-out” analysis for the pooled prevalence of PLWH in COVID-19 cases. The red vertical line represents the aggregate effect size when all studies were included in the meta-analysis. The dots represent the aggregate effect size when the study listed next to the dot was removed from the analysis; Figure S3: An influence plot from a “leave-one-out” analysis for the relative risk of severe COVID-19 in PLWH compared to HIV-negative patients. The red vertical line represents the aggregate effect size when all studies were included in the meta-analysis. The dots represent the aggregate effect size when the study listed next to the dot was removed from the analysis; Figure S4: An influence plot from a “leave-one-out” analysis for the relative risk of COVID-19 mortality in PLWH compared to HIV-negative patients. The red vertical line represents the aggregate effect size when all studies were included in the meta-analysis. The dots represent the aggregate effect size when the study listed next to the dot was removed from the analysis; Figure S5: Pooled prevalence of PLWH co-infected with SARS-CoV-2 among COVID-19 cases for Continental Asia alone. The red dotted line represents the overall effect size. The lateral edges of the blue diamond represent the limits of the 95% confidence intervals (ES: Effect size, CI: Confidence Interval).
