# Supplementary material for: Global and Regional Prevalence and Outcomes of COVID-19 in People Living with HIV: A Systematic Review and Meta-Analysis
Source: Trop Med Infect Dis. 2022 Feb 3;7(2):22. doi: 10.3390/tropicalmed7020022 (PMC8880028; doi:10.3390/tropicalmed7020022)
Supplement: Supplementary file 1 [file tropicalmed-07-00022-s001.zip › supplementary.pdf]

5Supplemental materials

Table S1 - Quality Assessment.

Author (publication year)	SELECTION				ASCERTAINMENT		OUTCOME			OVERALL SCORE (≥5 Stars = Lower Risk of Bias)
	Adequate sample size (>=100)	Sample representative of population (Multicentre)	Both groups drawn from same community	Randomness in recruitment	WHO-standard diagnosis (RT-PCR)	HIV Comorbidity confirmation (EMR/Test)	Outcome Reported Adequately (EMR)	Follow up duration adequate (>= 2 weeks (1))	All subjects accounted for	
Di Biagio et al (2020) (2)	*	*	*	-	*	*	*	-	*	7
Borobia et al (2020) (3)	*	-	*	-	-	*	*	-	*	6
Boulle et al (2020) (4)	*	*	*	*	*	*	*	*	-	8
Ceballos et al (2021) (5)	*	*	*	*	*	*	*	*	*	9
Collins et al (2020) (6)	*	*	*	*	*	*	*	-	*	7
Del Amo et al (2020) (7)	*	*	*	-	*	*	*	*	*	8
Docherty et al (2020) (8)	*	*	*	*	-	*	*	*	*	8
Erinoso et al (2020) (9)	*	*	*	*	*	*	*	*	*	9
Etienne et al (2020) (10)	-	-	-	-	-	*	*	*	*	4
Garetti et al (2020) (11)	*	*	*	*	*	*	*	*	*	9
Gervasoni et al (2020) (12)	-	-	*	-	-	*	*	*	-	4
Geteneh et al (2021) (13)	*	-	*	*	*	*	*	-	-	6
Gudipati et al (2020) (14)	*	-	*	-	-	*	*	-	-	4
Hadi et al (2020) (15)	*	-	*	-	-	*	*	*	-	5
Harter et al (2020) (16)	-	-	-	-	*	*	*	*	-	4
Ho et al (2020) (17)	-	*	*	-	-	*	*	-	*	5
Huang et al (2020) (18)	*	*	*	*	*	*	*	-	*	8
Inciarte et al (2020) (19)	*	-	*	*	*	*	*	-	*	7

Isernia et al (2020) (20)	-	-	-	-	-	*	*	*	*	4
Izquierdo et al (2020) (21)	*	*	*	*	*	*	*	-	*	8
Karim et al (2020) (22)	*	-	*	*	*	*	*	*	*	8
Kirenga et al (2020) (23)	-	-	*	*	*	*	*	*	*	7
Liu (2020) (24)	-	-	-	-	*	*	*	*	*	5
Maggiolo et al (2020) (25)	-	-	-	-	*	*	*	*	-	5
Migisha et al (2020) (26)	-	*	*	-	-	*	*	-	*	4
Miyashita & Kuno (2020) (27)	*	*	*	*	*	*	*	-	*	8
Nachega et al (2020) (28)	*	*	*	*	*	*	*	*	*	9
Ombajo et al (2020) (29)	*	*	*	*	*	*	*	-	*	8
Parker et al (2020) (30)	*	-	*	*	*	*	*	*	*	8
Pujari et al (2021) (31)	-	-	*	-	*	*	*	-	*	5
Rodriguez-Gonzalez et al (2021) (32)	*	-	*	*	*	*	*	*	*	8
Rodriguez-Molinero et al (2020) (33)	*	*	*	*	*	*	*	-	*	8
Shalev et al (2020) (34)	-	-	*	*	*	*	*	-	*	6
Shi et al (2020) (35)	*	-	*	*	*	*	*	*	*	8
Sigel et al (2020) (36)	-	*	*	*	-	*	*	*	*	7
Silver et al (2020) (37)	*	-	*	*	*	*	*	*	*	8
Stoeckle et al (2020) (38)	*	*	-	-	-	*	*	*	*	6
Tesoriero et al (2020) (39)	*	*	*	-	*	*	*	-	*	7
Virata et al (2020) (40)	*	-	*	-	-	*	*	*	*	6
Vizcarra et al (2020) (41)	*	-	*	-	*	*	*	*	*	7
Wang et al (2020) (42)	*	-	*	*	*	*	*	*	*	8
Yang et al (2021) (43)	*	-	*	*	*	*	*	*	-	7

Yu et al (2020) (44)	*	-	*	*	-	*	*	-	*	6

PCR, Polymerase Chain Reaction; EMR, Electronic Medical Record.

Database: Embase Classic+Embase <1947 to 2021 June 22>
Search Strategy:
----
1 exp COVID-19/ (123940)
2 exp Severe acute respiratory syndrome coronavirus 2/ (34233)
3 1 or 2 (128690)
4 exp Human immunodeficiency virus/ (204532)
5 3 and 4 (819)
6 limit 5 to (full text and human and english language) (82)

****

Database: Embase Classic+Embase <1947 to 2021 January 10>
Search Strategy:
----
1 Coronavirus Infections/ or COVID-19.mp. (65781)
2 2019 novel coronavirus.mp. (1305)
3 2019 coronavirus.mp. (344)
4 2019-nCoV.mp. (1337)
5 SARS-nCoV 2.mp. [mp=title, abstract, heading word, drug trade name, original title, device manufacturer, drug manufacturer, device trade name, keyword, floating subheading word, candidate term word] (14)
6 SARS-CoV-2.mp. (22727)
7 1 or 2 or 3 or 4 or 5 or 6 (70423)
8 clinical characteristic*.mp. [mp=title, abstract, heading word, drug trade name, original title, device manufacturer, drug manufacturer, device trade name, keyword, floating subheading word, candidate term word] (123413)
9 clinical characteristics.mp. (121781)
10 clinical feature*.mp. [mp=title, abstract, heading word, drug trade name, original title, device manufacturer, drug manufacturer, device trade name, keyword, floating subheading word, candidate term word] (771891)
11 clinical features.mp. (164456)
12 8 or 9 or 10 or 11 (854476)
13 7 and 12 (3667)
14 limit 13 to (english language and full text) (520)

Database: Ovid MEDLINE(R) and Epub Ahead of Print, In-Process & Other Non-Indexed Citations and Daily <1946 to November 20, 2020>
Search Strategy:
----
1 Coronavirus Infections/ or COVID-19.mp. (76612)
2 2019 novel coronavirus.mp. (1164)
3 2019 coronavirus.mp. (396)
4 2019-nCoV.mp. (1317)
5 SARS-nCoV 2.mp. [mp=title, abstract, original title, name of substance word, subject heading word, floating sub-heading word, keyword heading word, organism supplementary concept word, protocol supplementary concept word, rare disease supplementary concept word, unique identifier, synonyms] (14)
6 SARS-CoV-2.mp. (23541)
7 1 or 2 or 3 or 4 or 5 or 6 (79087)
8 clinical characteristic*.mp. [mp=title, abstract, original title, name of substance word, subject heading word, floating sub-heading word, keyword heading word, organism supplementary concept word, protocol supplementary concept word, rare disease supplementary concept word, unique identifier, synonyms] (73288)
9 clinical characteristics.mp. (72538)
10 clinical feature*.mp. [mp=title, abstract, original title, name of substance word, subject heading word, floating sub-heading word, keyword heading word, organism supplementary concept word, protocol supplementary concept word, rare disease supplementary concept word, unique identifier, synonyms] (109298)
11 clinical features.mp. (105432)
12 8 or 9 or 10 or 11 (176949)
13 7 and 12 (2196)
14 limit 13 to (english language and full text) (296)

****
Database: Ovid MEDLINE(R) ALL <1946 to June 23, 2021>
Search Strategy:
----
1 Coronavirus Infections/ or Coronavirus/ or COVID-19.mp. (147706)
2 novel coronavirus.mp. [mp=title, abstract, original title, name of substance word, subject heading word, floating sub-heading word, keyword heading word, organism supplementary concept word, protocol supplementary concept word, rare disease supplementary concept word, unique identifier, synonyms] (9046)
3 SARS-CoV-2.mp. [mp=title, abstract, original title, name of substance word, subject heading word, floating sub-heading word, keyword heading word, organism supplementary concept word, protocol supplementary concept word, rare disease supplementary concept word, unique identifier, synonyms] (91033)
4 2019-nCoV.mp. [mp=title, abstract, original title, name of substance word, subject heading word, floating sub-heading word, keyword heading word, organism supplementary concept word, protocol supplementary concept word, rare disease supplementary concept word, unique identifier, synonyms] (1725)
5 1 or 2 or 3 or 4 (152312)
6 clinical characteristics.mp. (78474)
7 clinical characteristic*.mp. [mp=title, abstract, original title, name of substance word, subject heading word, floating sub-heading word, keyword heading word, organism supplementary concept word, protocol supplementary concept word, rare disease supplementary concept word, unique identifier, synonyms] (79277)
8 clinical features.mp. (110348)
9 clinical feature*.mp. [mp=title, abstract, original title, name of substance word, subject heading word, floating sub-heading word, keyword heading word, organism supplementary concept word, protocol supplementary concept word, rare disease supplementary concept word, unique identifier, synonyms] (114354)
10 6 or 7 or 8 or 9 (187432)
11 5 and 10 (3801)
12 exp HIV/ or exp Anti-HIV Agents/ or HIV-2/ or exp HIV Infections/ or exp HIV-1/ (342413)
13 exp Acquired Immunodeficiency Syndrome/ (76800)
14 12 or 13 (342413)
15 11 and 14 (60)
16 limit 15 to "review articles" (3)
17 15 not 16 (57)
18 limit 17 to english language (54)

****

Figure S1 – Search Strategy

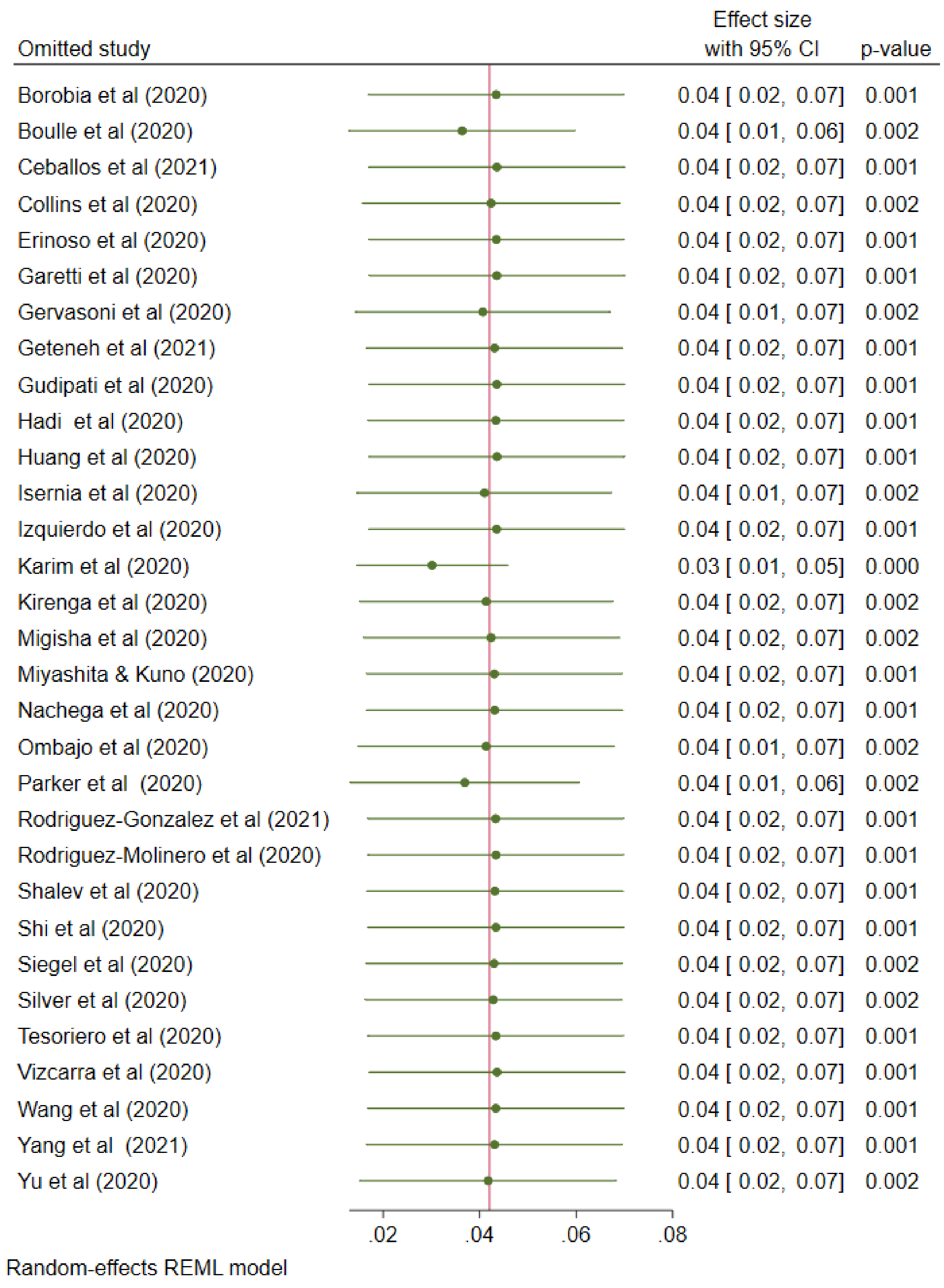

Figure S2. An influence plot from a "leave-one-out" analysis for the pooled prevalence of PLWH in COVID-19 cases. The red vertical line represents the aggregate effect size when all studies were included in the meta-analysis. The dots represent the aggregate effect size when the study listed next to the dot was removed from the analysis.

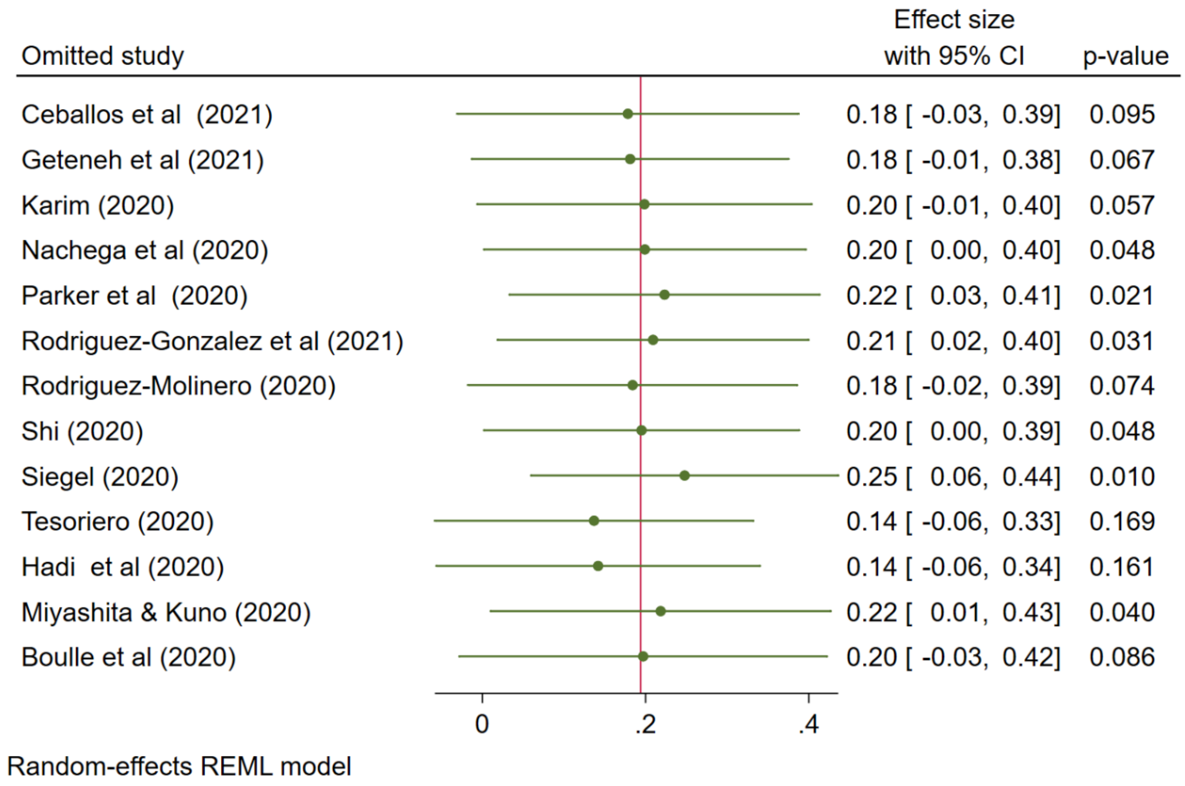

Figure S3. An influence plot from a "leave-one-out" analysis for the relative risk of severe COVID-19 in PLWH compared to HIV-negative patients. The red vertical line represents the aggregate effect size when all studies were included in the meta-analysis. The dots represent the aggregate effect size when the study listed next to the dot was removed from the analysis.

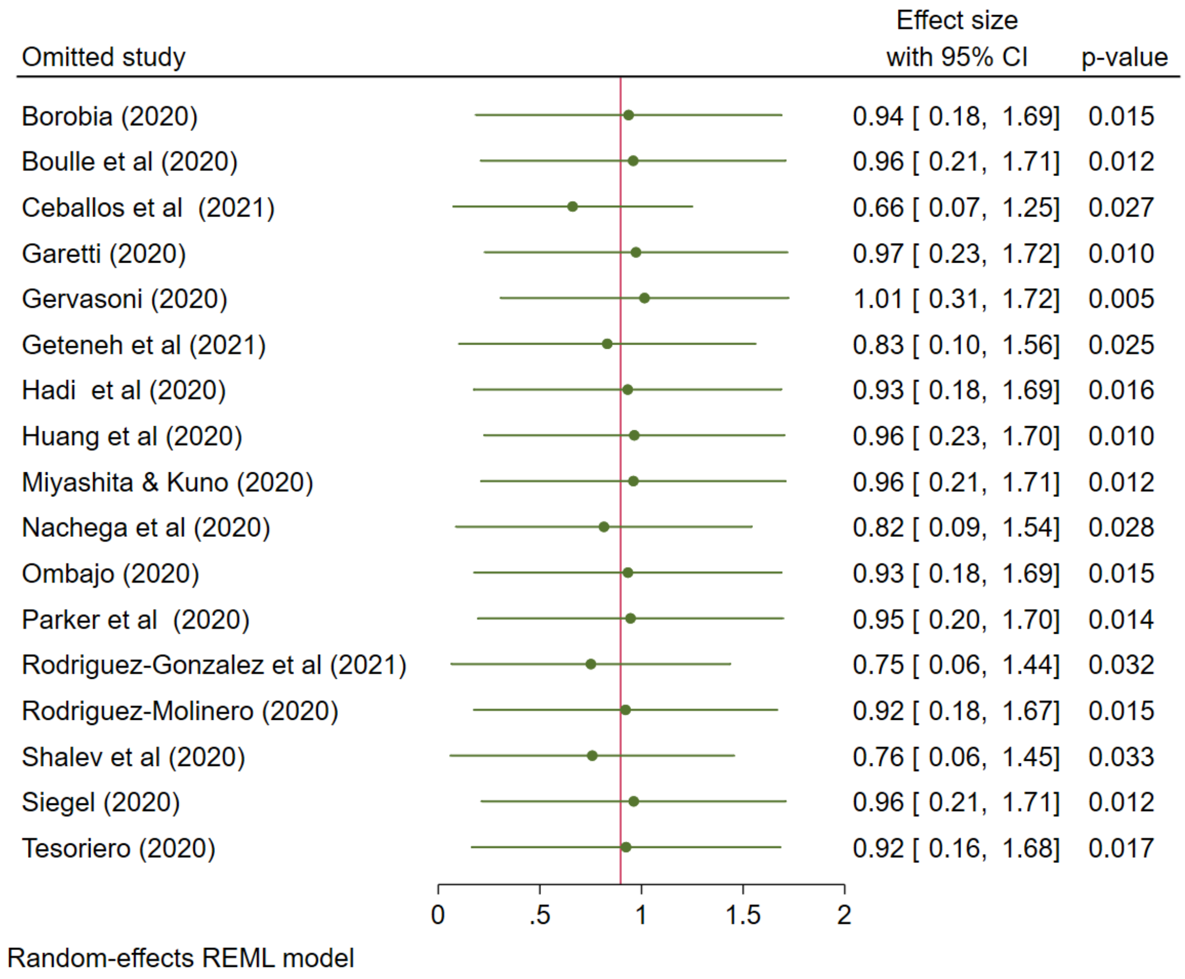

Figure S4. An influence plot from a "leave-one-out" analysis for the relative risk of COVID-19 mortality in PLWH compared to HIV-negative patients. The red vertical line represents the aggregate effect size when all studies were included in the meta-analysis. The dots represent the aggregate effect size when the study listed next to the dot was removed from the analysis.

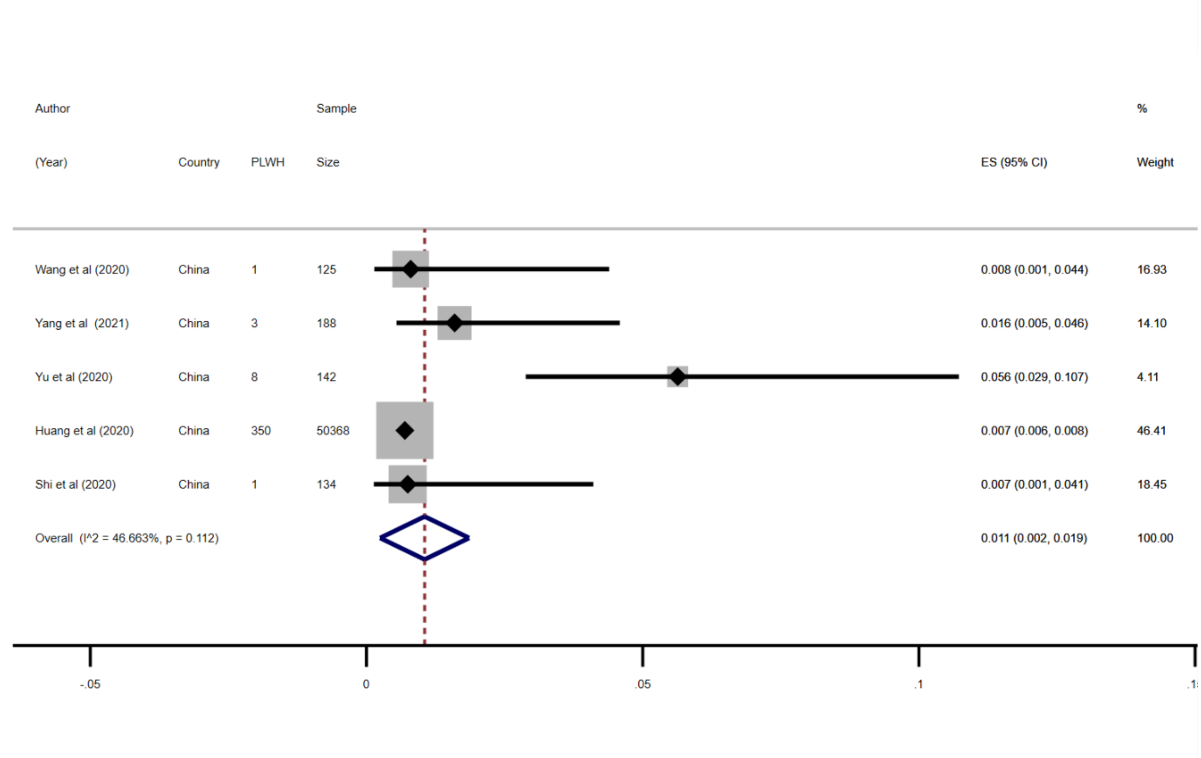

Figure S5: Pooled prevalence of PLWH co-infected with SARS-CoV-2among COVID-19 cases for Continental Asia alone. The red dotted line represents the overall effect size. The lateral edges of the blue diamond represent the limits of the 95% confidence intervals (ES: Effect size, CI: Confidence Interval).

PRISMA Checklist

Section and Topic	Item #	Checklist item	Location where item is reported
**TITLE**			
Title	1	Identify the report as a systematic review.	1
**ABSTRACT**			

Section and Topic	Item #	Checklist item	Location where item is reported
Abstract	2	Summary of study	2
**INTRODUCTION**			
Rationale	3	Describe the rationale for the review in the context of existing knowledge.	3-4
Objectives	4	Provide an explicit statement of the objective(s) or question(s) the review addresses.	4
**METHODS**			
Eligibility criteria	5	Specify the inclusion and exclusion criteria for the review and how studies were grouped for the syntheses.	5
Information sources	6	Specify all databases, registers, websites, organisations, reference lists and other sources searched or consulted to identify studies. Specify the date when each source was last searched or consulted.	5
Search strategy	7	Present the full search strategies for all databases, registers and websites, including any filters and limits used.	5 (Figure S1)
Selection process	8	Specify the methods used to decide whether a study met the inclusion criteria of the review, including how many reviewers screened each record and each report retrieved, whether they worked independently, and if applicable, details of automation tools used in the process.	5
Data collection process	9	Specify the methods used to collect data from reports, including how many reviewers collected data from each report, whether they worked independently, any processes for obtaining or confirming data from study investigators, and if applicable, details of automation tools used in the process.	5-6
Data items	10a	List and define all outcomes for which data were sought. Specify whether all results that were compatible with each outcome domain in each study were sought (e.g. for all measures, time points, analyses), and if not, the methods used to decide which results to collect.	6
	10b	List and define all other variables for which data were sought (e.g. participant and intervention characteristics, funding sources). Describe any assumptions made about any missing or unclear information.	6
Study risk of bias assessment	11	Specify the methods used to assess risk of bias in the included studies, including details of the tool(s) used, how many reviewers assessed each study and whether they worked independently, and if applicable, details of automation tools used in the process.	6-7
Effect measures	12	Specify for each outcome the effect measure(s) (e.g. risk ratio, mean difference) used in the synthesis or presentation of results.	6
Synthesis methods	13a	Describe the processes used to decide which studies were eligible for each synthesis (e.g. tabulating the study intervention characteristics and comparing against the planned groups for each synthesis (item #5)).	6
	13b	Describe any methods required to prepare the data for presentation or synthesis, such as handling of missing summary statistics, or data conversions.	NA
	13c	Describe any methods used to tabulate or visually display results of individual studies and syntheses.	6
	13d	Describe any methods used to synthesize results and provide a rationale for the choice(s). If meta-analysis was performed, describe the model(s), method(s) to identify the presence and extent of statistical heterogeneity, and software package(s) used.	6

Section and Topic	Item #	Checklist item	Location where item is reported
	13e	Describe any methods used to explore possible causes of heterogeneity among study results (e.g. subgroup analysis, meta-regression).	6
	13f	Describe any sensitivity analyses conducted to assess robustness of the synthesized results.	6
Reporting bias assessment	14	Describe any methods used to assess risk of bias due to missing results in a synthesis (arising from reporting biases).	6-7
Certainty assessment	15	Describe any methods used to assess certainty (or confidence) in the body of evidence for an outcome.	6
**RESULTS**			
Study selection	16a	Describe the results of the search and selection process, from the number of records identified in the search to the number of studies included in the review, ideally using a flow diagram.	7
	16b	Cite studies that might appear to meet the inclusion criteria, but which were excluded, and explain why they were excluded.	8
Study characteristics	17	Cite each included study and present its characteristics.	8
Risk of bias in studies	18	Present assessments of risk of bias for each included study.	8
Results of individual studies	19	For all outcomes, present, for each study: (a) summary statistics for each group (where appropriate) and (b) an effect estimate and its precision (e.g. confidence/credible interval), ideally using structured tables or plots.	9-11
Results of syntheses	20a	For each synthesis, briefly summarise the characteristics and risk of bias among contributing studies.	Table S1
	20b	Present results of all statistical syntheses conducted. If meta-analysis was done, present for each the summary estimate and its precision (e.g. confidence/credible interval) and measures of statistical heterogeneity. If comparing groups, describe the direction of the effect.	12-14
	20c	Present results of all investigations of possible causes of heterogeneity among study results.	12-14
	20d	Present results of all sensitivity analyses conducted to assess the robustness of the synthesized results.	Figure S2(a&b)
Reporting biases	21	Present assessments of risk of bias due to missing results (arising from reporting biases) for each synthesis assessed.	NA
Certainty of evidence	22	Present assessments of certainty (or confidence) in the body of evidence for each outcome assessed.	12-14
**DISCUSSION**			
Discussion	23a	Provide a general interpretation of the results in the context of other evidence.	15-18
	23b	Discuss any limitations of the evidence included in the review.	18-19
	23c	Discuss any limitations of the review processes used.	18-19
	23d	Discuss implications of the results for practice, policy, and future research.	19
**OTHER INFORMATION**			
Registration and protocol	24a	Provide registration information for the review, including register name and registration number, or state that the review was not registered.	5
	24b	Indicate where the review protocol can be accessed, or state that a protocol was not prepared.	5
	24c	Describe and explain any amendments to information provided at registration or in the protocol.	NA

Section and Topic	Item #	Checklist item	Location where item is reported
Support	25	Describe sources of financial or non-financial support for the review, and the role of the funders or sponsors in the review.	NA
Competing interests	26	Declare any competing interests of review authors.	NA
Availability of data, code and other materials	27	Report which of the following are publicly available and where they can be found: template data collection forms; data extracted from included studies; data used for all analyses; analytic code; any other materials used in the review.	NA

References

1. Organization WH. COVID-19 clinical management: living guidance, 25 January 2021. World Health Organization; 2021.
2. Di Biagio A, Ricci E, Calza L, Squillace N, Menzaghi B, Rusconi S, et al. Factors associated with hospital admission for COVID-19 in HIV patients. Aids. 2020;34(13):1983-5.
3. Borobia AM, Carcas AJ, Arnalich F, Álvarez-Sala R, Monserrat-Villatoro J, Quintana M, et al. A Cohort of Patients with COVID-19 in a Major Teaching Hospital in Europe. J Clin Med. 2020;9(6).
4. Boulle A, Davies MA, Hussey H, Ismail M, Morden E, Vundle Z, et al. Risk factors for COVID-19 death in a population cohort study from the Western Cape Province, South Africa. Clin Infect Dis. 2020.
5. Ceballos ME, Ross P, Lasso M, Dominguez I, Puente M, Valenzuela P, et al. Clinical characteristics and outcomes of people living with HIV hospitalized with COVID-19: a nationwide experience. International journal of STD & AIDS. 2021;32(5):435-43.
6. Collins LF, Moran CA, Oliver NT, Moanna A, Lahiri CD, Colasanti JA, et al. Clinical characteristics, comorbidities and outcomes among persons with HIV hospitalized with coronavirus disease 2019 in Atlanta, Georgia. AIDS (London, England). 2020;34(12):1789.
7. Del Amo J, Polo R, Moreno S, Díaz A, Martínez E, Arribas JR, et al. Incidence and Severity of COVID-19 in HIV-Positive Persons Receiving Antiretroviral Therapy : A Cohort Study. Ann Intern Med. 2020;173(7):536-41.
8. Docherty AB, Harrison EM, Green CA, Hardwick HE, Pius R, Norman L, et al. Features of 20 133 UK patients in hospital with covid-19 using the ISARIC WHO Clinical Characterisation Protocol: prospective observational cohort study. Bmj. 2020;369:m1985.
9. Erinoso OA, Wright KO, Anya S, Bowale A, Adejumo O, Adesola S, et al. Clinical characteristics, predictors of symptomatic coronavirus disease 2019 and duration of hospitalisation in a cohort of 632 Patients in Lagos State, Nigeria. Niger Postgrad Med J. 2020;27(4):285-92.
10. Etienne N, Karmochkine M, Slama L, Pavie J, Batisse D, Usubillaga R, et al. HIV infection and COVID-19: risk factors for severe disease. Aids. 2020;34(12):1771-4.
11. Geretti AM, Stockdale AJ, Kelly SH, Cevik M, Collins S, Waters L, et al. Outcomes of COVID-19 related hospitalization among people with HIV in the ISARIC WHO Clinical Characterization Protocol (UK): a prospective observational study. Clin Infect Dis. 2020.
12. Gervasoni C, Meraviglia P, Riva A, Giacomelli A, Oreni L, Minisci D, et al. Clinical Features and Outcomes of Patients With Human Immunodeficiency Virus With COVID-19. Clin Infect Dis. 2020;71(16):2276-8.
13. Geteneh A, Alemnew B, Tadesse S, Girma A. Clinical characteristics of patients infected with SARS-CoV-2 in North Wollo Zone, North-East Ethiopia. Pan Afr Med J. 2021;38:217.
14. Gudipati S, Brar I, Murray S, McKinnon JE, Yared N, Markowitz N. Descriptive Analysis of Patients Living With HIV Affected by COVID-19. JAIDS Journal of Acquired Immune Deficiency Syndromes. 2020;85(2).
15. Hadi YB, Naqvi SF, Kupec JT, Sarwari AR. Characteristics and outcomes of COVID-19 in patients with HIV: a multicentre research network study. Aids. 2020;34(13):F3-F8.
16. Härter G, Spinner CD, Roider J, Bickel M, Krznaric I, Grunwald S, et al. COVID-19 in people living with human immunodeficiency virus: a case series of 33 patients. Infection. 2020:1.
17. Ho H-e, Peluso MJ, Margus C, Matias Lopes JP, He C, Gaisa MM, et al. Clinical outcomes and immunologic characteristics of coronavirus disease 2019 in people with human immunodeficiency virus. The Journal of infectious diseases. 2021;223(3):403-8.
18. Huang Y, Jiang Y, Li Z, Han D, Wu L, Ma J, et al. Comparison of initial high-resolution computed tomography (HRCT) features of coronavirus disease 2019 (COVID-19) pneumonia and other viral pneumonias. Annals of Palliative Medicine. 2021;10(1):560-71.
19. Inciarte A, Gonzalez-Cordon A, Rojas J, Torres B, de Lazzari E, de la Mora L, et al. Clinical characteristics, risk factors, and incidence of symptomatic coronavirus disease 2019 in a large cohort

of adults living with HIV: a single-center, prospective observational study. AIDS (London, England). 2020;34(12):1775.
20. Isernia V, Julia Z, Le Gac S, Bachelard A, Landman R, Lariven S, et al. SARS-COV2 infection in 30 HIV-infected patients followed-up in a French University Hospital. International Journal of Infectious Diseases. 2020;101:49-51.
21. Izquierdo JL, Ancochea J, Soriano JB, Group SC-R. Clinical characteristics and prognostic factors for intensive care unit admission of patients With COVID-19: retrospective study using machine learning and natural language processing. Journal of medical Internet research. 2020;22(10):e21801.
22. Karim F, Gazy I, Cele S, Zungu Y, Krause R, Bernstein M, et al. HIV infection alters SARS-CoV-2 responsive immune parameters but not clinical outcomes in COVID-19 disease. medRxiv. 2020.
23. Kirenga B, Muttamba W, Kayongo A, Nsereko C, Siddharthan T, Lusiba J, et al. Characteristics and outcomes of admitted patients infected with SARS-CoV-2 in Uganda. BMJ open respiratory research. 2020;7(1):e000646.
24. Liu J, Zeng W, Cao Y, Cui Y, Li Y, Yao S, et al. Effect of a previous history of antiretroviral treatment on the clinical picture of patients with co-infection of SARS-CoV-2 and HIV: A preliminary study. International Journal of Infectious Diseases. 2020;100:141-8.
25. Maggiolo F, Zoboli F, Arosio M, Valenti D, Guarneri D, Sangiorgio L, et al. SARS-CoV-2 infection in persons living with HIV: a single center prospective cohort. Journal of medical virology. 2021;93(2):1145-9.
26. Migisha R, Kwesiga B, Mirembe BB, Amanya G, Kabwama SN, Kadobera D, et al. Early cases of SARS-CoV-2 infection in Uganda: epidemiology and lessons learned from risk-based testing approaches–March-April 2020. Globalization and Health. 2020;16(1):1-9.
27. Miyashita H, Kuno T. Prognosis of coronavirus disease 2019 (COVID-19) in patients with HIV infection in New York City. HIV Med. 2021;22(1):e1-e2.
28. Nachega JB, Ishoso DK, Otokoye JO, Hermans MP, Machekano RN, Sam-Agudu NA, et al. Clinical Characteristics and Outcomes of Patients Hospitalized for COVID-19 in Africa: Early Insights from the Democratic Republic of the Congo. Am J Trop Med Hyg. 2020;103(6):2419-28.
29. Ombajo LA, Mutono N, Sudi P, Mutua M, Sood M, Ali Loo AM, et al. EPIDEMIOLOGICAL AND CLINICAL CHARACTERISTICS OF COVID-19 PATIENTS IN KENYA. medRxiv. 2020:2020.11.09.20228106.
30. Parker A, Koegelenberg CFN, Moolla MS, Louw EH, Mowlana A, Nortjé A, et al. High HIV prevalence in an early cohort of hospital admissions with COVID-19 in Cape Town, South Africa2020.
31. Pujari S, Gaikwad S, Chitalikar A, Dabhade D, Joshi K, Bele V. Coronavirus Disease 19 Among People Living with HIV in Western India: An Observational Cohort Study. AIDS Research and Human Retroviruses. 2021.
32. Rodriguez-Gonzalez CG, Chamorro-de-Vega E, Valerio M, Amor-Garcia MA, Tejerina F, Sancho-Gonzalez M, et al. COVID-19 in hospitalised patients in Spain: a cohort study in Madrid. International Journal of Antimicrobial Agents. 2021;57(2):106249.
33. Rodríguez-Molinero A, Gálvez-Barrón C, Miñarro A, Macho O, López GF, Robles MT, et al. Association between COVID-19 prognosis and disease presentation, comorbidities and chronic treatment of hospitalized patients. PLOS ONE. 2020;15(10):e0239571.
34. Shalev N, Scherer M, LaSota ED, Antoniou P, Yin MT, Zucker J, et al. Clinical Characteristics and Outcomes in People Living With Human Immunodeficiency Virus Hospitalized for Coronavirus Disease 2019. Clin Infect Dis. 2020;71(16):2294-7.
35. Shi P, Ren G, Yang J, Li Z, Deng S, Li M, et al. Clinical characteristics of imported and second-generation coronavirus disease 2019 (COVID-19) cases in Shaanxi outside Wuhan, China: a multicentre retrospective study. Epidemiology and Infection. 2020;148:e238.
36. Sigel K, Swartz T, Golden E, Paranjpe I, Somani S, Richter F, et al. Coronavirus 2019 and People Living With Human Immunodeficiency Virus: Outcomes for Hospitalized Patients in New York City. Clin Infect Dis. 2020;71(11):2933-8.

37. Silver V, Chapple AG, Feibus AH, Beckford J, Halapin NA, Barua D, et al. Clinical Characteristics and Outcomes Based on Race of Hospitalized Patients With COVID-19 in a New Orleans Cohort. Open Forum Infect Dis. 2020;7(9):ofaa339.
38. Stoeckle K, Johnston CD, Jannat-Khah DP, Williams SC, Ellman TM, Vogler MA, et al. COVID-19 in Hospitalized Adults With HIV. Open Forum Infect Dis. 2020;7(8):ofaa327.
39. Tesoriero JM, Swain C-AE, Pierce JL, Zamboni L, Wu M, Holtgrave DR, et al. COVID-19 Outcomes Among Persons Living With or Without Diagnosed HIV Infection in New York State. JAMA Network Open. 2021;4(2):e2037069-e.
40. Virata MD, Shenoi S, Ladines-Lim JB, Villanueva M, Aoun-Barakat L. 111. Outcomes Related to COVID-19 Among People Living with HIV: Cohort from a Large Academic Center: Open Forum Infect Dis. 2020 Dec 31;7(Suppl 1):S184. doi: 10.1093/ofid/ofaa439.421. eCollection 2020 Oct.
41. Vizcarra P, Pérez-Elías MJ, Quereda C, Moreno A, Vivancos MJ, Dronda F, et al. Description of COVID-19 in HIV-infected individuals: a single-centre, prospective cohort. Lancet HIV. 2020;7(8):e554-e64.
42. Wang R, Pan M, Zhang X, Han M, Fan X, Zhao F, et al. Epidemiological and clinical features of 125 Hospitalized Patients with COVID-19 in Fuyang, Anhui, China. Int J Infect Dis. 2020;95:421-8.
43. Yang R, Gui X, Zhang Y, Xiong Y, Gao S, Ke H. Clinical characteristics of COVID-19 patients with HIV coinfection in Wuhan, China. Expert Review of Respiratory Medicine. 2021;15(3):403-9.
44. Yu Y, Tu J, Lei B, Shu H, Zou X, Li R, et al. Incidence and Risk Factors of Deep Vein Thrombosis in Hospitalized COVID-19 Patients. Clin Appl Thromb Hemost. 2020;26:1076029620953217.
